# Genome sequence of antibiotic-resistant Klebsiella quasipneumoniae FSFC0558: a novel sequence type (ST8212)

**DOI:** 10.1099/acmi.0.000944.v3

**Published:** 2025-04-25

**Authors:** Daniel Robins, Richard N. Goodman, Ralfh Pulmones, Upendo O. Kibwana, Joel Manyahi, Bjørn Blomberg, Nina Langeland, Sabrina Moyo, Adam P. Roberts

**Affiliations:** 1 Department of Tropical Disease Biology, Liverpool School of Tropical Medicine, Liverpool, UK; 2 Department of Microbiology and Immunology, Muhimbili University of Health and Allied Sciences, Dar es Salaam, Tanzania; 3 Department of Clinical Science, University of Bergen, Bergen, Norway; 4 Norwegian National Advisory Unit on Tropical Infectious Diseases, Haukeland University Hospital, Bergen, Norway

**Keywords:** antibiotic resistance, genome sequence, *Klebsiella quasipneumoniae*, sepsis

## Abstract

*Klebsiella quasipneumoniae* are Gram-negative bacteria of the family *Enterobacteriaceae*, distinguished from other members of the *Klebsiella* genus through a chromosomally encoded extended spectrum β-lactamase, *bla_OKP_*. Here, we report a hybrid assembled genome of a novel sequence type of *K. quasipneumoniae* subspecies *similipneumoniae* isolated from a faecal sample of a patient with sepsis.

## Data Summary

Whole-genome sequence (WGS) data are accessible through bioproject number PRJNA1154671 under accession numbers CP171744.1 (chromosome) and CP171745.1 (plasmid). The WGS data was uploaded to the *Klebsiella* PasteurMLST database (id=76021) and designated as a new sequence type: ST8212.

## Announcement

*Klebsiella* have evolved rapidly, with the differentiation of *K. quasipneumoniae* in 2014 [1]. Multidrug-resistant, extended spectrum β-lactamase (ESBL)-producing and hypervirulent *K. quasipneumoniae* strains have been isolated from hospital-acquired and community-acquired infections globally [2,3]. However, misidentification as *K. pneumoniae* is common, hindering medical interventions and research progress [2]. *K. quasipneumoniae* comprises 2–10% of *Klebsiella* infections, with increasing prevalences of serious *K. quasipneumoniae* infections becoming a concern [2,4, 5].

The strain FSFC0558 was collected in a previous study in Tanzania in October 2017 [6,7]. The strain was isolated from a rectal swab of a neonate and screened for ESBLs using the selective media, ChromID ESBL. The bacterial strain was identified by MALDI-TOF and tested for phenotypic antimicrobial susceptibility by disc diffusion as described previously [7] in accordance with guidelines and breakpoints of the Clinical Laboratory Standards Institute [8]. The isolate was stored at −70 °C at the Liverpool School of Tropical Medicine, UK.

For short-read sequencing, the strain was cultured and processed according to MicrobesNG’s strain submission guidelines (https://microbesng.com/). Illumina sequencing was performed by MicrobesNG (2×250 bp paired-end reads). Nanopore sequencing was carried out using SQK-NBD114.24 Ligation Sequencing and Native Barcoding Kit and an FLO-MIN114 (R10.4.1) flow cell (Oxford Nanopore Technologies, Oxford, UK) on a MinION Mk1B sequencer. Long-read data were base-called using guppy v6.4.2 with the super accuracy (sup) model. Read quality was assessed using FastQC [9] and Nanoplot v1.38.1 [10]. The *de novo* hybrid assembly used hybracter v0.6.0 [11].

Antimicrobial resistance (AMR) genes were determined using Resfinder v4.6.0 [12], defining the genus as *Klebsiella* with default parameters (60% coverage and 90% sequence identity) (Table 1). Virulence genes were determined using abricate [13] and the virulence factor database [14] with thresholds above 90% coverage and 90% sequence identity. Multi-locus sequence typing (MLST) v2.0 determined a novel sequence type (ST), species selected as *Klebsiella pneumoniae* (Table 1) [15]. The presence of chromosomally encoded *bla_OKP-B-8_* distinguished the species as *Klebsiella quasipneumoniae* subsp. *similipneumoniae*. The K-type is K124 and the O-type is O3/O3a.

**Table 1.** Genomic analysis of novel ST *K. quasipneumoniae* subsp. s*imilipneumoniae* (KpII-B)

Species		Reads				Assembly statistics			Contigs					
Species	MLST	Raw reads	Trimmed reads	Read depth	Read N50 (bp)	Assemblytotal length	Assembly total GC%	Bioproject no.	Contig type	Total length (bp)	GC (%)	Plasmid replicons	Virulence genes	AMR genes
*K. quasipneumoniae* subsp. *similipneumoniae*	Novel	N/A (I)	3,945,380 (I)	136 (I)	250 (I)	5,269,471	58.02	^PRJNA1154671^	Chromosome	5,102,762	58.22	n/a	yagZ/ecpA	blaOKP-B-8, oqxAB, fosA
									Plasmid	166,709	52.09	IncFII(K)	n/a	aac(3)-lld, blaCTX-M-15, blaTEM-1B, dfrA30
		580,218 (O)	195,788 (O)	222 (O)	13,392 (O)									

I, Illumina; O, Oxford Nanopore Technologies; N/A, not applicable.

The isolate was resistant to aztreonam, ceftazidime and gentamicin but susceptible to ciprofloxacin [7]. The phenotypic resistance aligns to the genotypic resistance as it carries the aztreonam and ceftazidime resistance gene *bla*_CTX-M-15_, and the gentamicin resistance gene *aac(3)-IId*. However, the susceptibility is genotypically and phenotypically mismatched, as the isolate carries the *oqxAB* genes which can confer resistance to ciprofloxacin even though it is phenotypically susceptible.

A nonsynonymous substitution was identified in the *mdh55* gene encoding a malate dehydrogenase, responsible for catalysing oxaloacetate into malate [16]. The whole-genome sequence and *mdh55* gene outputs were analysed with blast [17]. There have been no previous reports of *mdh55* with this mutation; therefore, we identified a novel ST. Subsequently, we have added this sequence to the *Klebsiella* PasteurMLST database, which has been designated as a new sequence type: ST8212.

This genome sequence will provide a reference for the continued tracking of this *K. quasipneumoniae* ST as the identification of novel STs remains crucially important for understanding evolutionary trajectories at the strain level and epidemiological changes at larger population and geographical scales.

## Abbreviations

AMR: antimicrobial resistance
ESBL: extended spectrum β-lactamase
MLST: multi-locus sequence typing
ST: sequence type
WGS: whole-genome sequence
